# Making complex decisions in uncertain times: experiences of Dutch GPs as gatekeepers regarding hospital referrals during COVID-19—a qualitative study

**DOI:** 10.1186/s12910-021-00725-0

**Published:** 2021-11-30

**Authors:** Dieke Westerduin, Janneke Dujardin, Jaap Schuurmans, Yvonne Engels, Anne B. Wichmann

**Affiliations:** Department of Anesthesiology, Pain and Palliative Medicine, Radboud University Medical Center, Nijmegen, The Netherlands

**Keywords:** Qualitative research, Ethical issues, Sars coronavirus, General practitioners, Hospital referral, Shared decision making

## Abstract

**Background:**

General practitioners often act as gatekeeper, authorizing patients’ access to hospital care. This gatekeeping role became even more important during the current COVID-19 crisis as uncertainties regarding COVID-19 made estimating the desirability of hospital referrals (for outpatient or inpatient hospitalization) complex, both for COVID and non-COVID suspected patients. This study explored Dutch general practitioners’ experiences and ethical dilemmas faced in decision making about hospital referrals in times of the COVID-19 pandemic.

**Methods:**

Semi-structured interviews with Dutch general practitioners working in the Netherlands were conducted. Participants were recruited via purposive sampling. Thematic analysis was conducted using content coding.

**Results:**

Fifteen interviews were conducted, identifying four themes: one overarching regarding (1) COVID-19 uncertainties, and three themes about experienced ethical dilemmas: (2) the patients’ self-determination vs. the general practitioners’ paternalism, (3) the general practitioners’ duty of care vs. the general practitioners’ autonomy rights, (4) the general practitioners’ duty of care vs. adequate care provision.

**Conclusions:**

Lack of knowledge about COVID-19, risks to infect loved ones, scarcity of hospital beds and loneliness of patients during hospital admission were central in dilemmas experienced. When developing guidelines for future crises, this should be taken into account.

## Introduction

In many countries, general practitioners (GPs) act as gatekeepers, authorizing patients’ access to hospital care. That is, for a patient to receive specialized hospital care, referral by his or her GP is needed [1, 2]. This gatekeeping role became even more important due to the current Corona Virus Disease 2019 (COVID-19) crisis, both for COVID and non-COVID suspected patients. Due to COVID-19, healthcare systems face several challenges [3–6]. Particularly intensive care unit (ICU) resources are scarce, making regional, national and international collaboration necessary [7, 8]. More specifically, in the Netherlands a GP referral is needed for visits to hospitals [9]. The Netherlands had a pandemic plan in order for GPs to assess all potential COVID-19 patients. GPs were able to reorganise their flow of patients from mainly face-to-face to virtual consultations, and separated practice visits of suspected from non-COVID patients [10]. There has been documented decreases in the use of GP services, assumedly due to the already overstretched health care system, a perceived increased risk of COVID-19 exposure in healthcare settings, and directives to self-isolate at home [10]. Although hospital referral always has to be carefully considered, as it confronts a patient with an interplay of stressors [11], hospitalization during COVID-19 has far-reaching consequences. Patients are even more socially deprived than during usual admissions, as visitors are not or only limited allowed. Such isolation can cause loneliness, anxiety, depression and a delirium [12].

As COVID-19 concerns a previously unknown disease, it is difficult to estimate for whom hospital referral would be beneficial or harmful [13]. In shared decision making (SDM), information is provided and patients are supported to consider and articulate their preferences and views during the decision making process [14]. It increases patient knowledge, confidence in decisions, and preference for more conservative treatment options [15, 16], and provides an approach to discuss Advance Care Planning (ACP) in a participative and informed manner, embodying the principles of person-centered care. Internationally, COVID-19 guidelines encourage general practice to start these discussions with their patients [17–20], including wishes regarding potential referral. Although GPs consider it an important aspect of their job, they also find such conversations difficult [21, 22]. Dealing with uncertainties about the prognosis, discussing future deterioration or death and taking the patient’s wishes into account make ACP conversations and proactive care planning difficult for GPs [23, 24].

During the COVID-19 crisis, this competency is even more challenged since it concerns a disease about which little is known. These uncertainties bring about several ethical concerns and dilemmas [25], such as the effects of communicating uncertainties on patient autonomy and well-being [26]. A recent paper therefore called upon the ethics community to start empirical research as to learn from front-line stakeholders about their experiences [27]. The aim of this study was to explore GPs’ experiences regarding their role as a gatekeeper in uncertain times, and which ethical dilemmas they face when making decisions about hospital referrals in times of the COVID-19 pandemic, both for COVID and non-COVID suspected patients.

## Methods

### Design

A qualitative research design was chosen, as the goal was to understand experiences and views of GPs, and obtain insights in dilemmas they faced [28]. An interpretative approach was used, in which the focus is understanding the world as others experience it [29]. Semi-structured interviews with GPs were conducted.

### Participants

GPs working in the Netherlands were recruited via the research teams’ networks and by contacting practices of which the email address was available on the internet. Exclusion criterion was not having practiced during the COVID-19 period. GPs working in both “clean” and “COVID-suspected” practices were interviewed. Purposive sampling was employed based on gender, work experience, geographical region of the practice and the number of COVID-19 patients in the practice. Participants were recruited via an invitation letter which was sent by email. One week later, GPs were contacted via telephone to ask if they wanted to participate. Recruitment stopped after data saturation was confirmed in five interviews in a row.

#### Data-collection

A topic guide of open ended questions was used, developed by the research team and based on earlier literature [21, 30]. See Appendix 1. The questions covered several aspects of possible experiences of the interviewees, e.g. regarding challenges and opportunities of COVID-19 with regard to decision making. Besides, moral considerations of the interviewees were covered in the topic list. Two pilot interviews were arranged, in which one or two researchers with considerable experience in interviewing participated (YE, AW, JS). Based on the pilot interviews, the topic list was finalized. Subsequently, each interview was conducted by two researchers (DW, JD) who had extensive communication and interview training, with a specific focus on person-centeredness. Table 1 shows characteristics of participating researchers. Because of governmental COVID-19 safety guidelines, all but one were conducted via video calling. After receiving informed consent, the interview was audio-recorded and transcribed verbatim. Field notes were made during the interview. The interviews took place between June and August 2020, and lasted 45–60 min each.

**Table 1:** Characteristics of researchers involved

Code	Initials	Gender	Age	Experience
I1	H.D.W	Female	23	Master student Medical sciences, Master student Philosophy. Experience in patient care. Limited experience in conducting interviews
I2	J.D	Female	24	Master student Medical sciences. Experience in patient care. Limited experience in conducting interviews
	J.P.S	Male	59	PhD student and active GP
	Y.E	Female	63	Professor in meaningful health care
	A.B.W	Female	33	Postdoctoral researcher in palliative care

### Analysis

Interviews were transcribed verbatim with the help of F4 software. Thematic content analysis with an open-coding scheme was performed. Firstly, transcripts were coded line-by-line using Atlas.ti software version 8.4.20. The first five interviews were coded independently by two researchers (DW, JD). After each interview, the coding was discussed until consensus was reached. The existing codebook was used as start for coding each next interview. After the process of initial coding, axial coding took place and codes were combined into categories and themes [31]. These were discussed and reviewed with the researcher team (YE, AW, JS, DW, JD). After this process, themes were defined.

### Ethics

Methods were carried out in accordance with relevant principles of the GCP guideline.

Protocols were checked by the Medical Review Ethics Committee region Arnhem-Nijmegen, which concluded this study was not subject to the Medical Research Involving Human Subjects Act (2020-6669) as participants were not subject to treatment, nor were they required to behave in a particular way. Informed consent was obtained from all subjects involved in the study.

## Results

Data saturation was reached after ten and confirmed in five additional interviews, resulting in a total of fifteen interviews, including the pilot interviews. Table 2 shows demographics of the participants. Where known, reasons for non-participation included lack of time or experience with hospital referrals during the COVID-19 pandemic. Fourteen interviews were conducted by phone, one face-to-face. Four main themes were identified. The first theme overarches the others.

**Table 2:** Characteristics of interviewees

Code	Gender	Age range	Years of experience	Other information
R1	Male	35–40	10	
R2	Female	30–35	4	Acting GP in two practices
R3	Female	30–35	1.5	Risk group
R4	Male	60–65	33	
R5	Female	40–45	14	Large number of elderly patients
R6	Male	40–45	10	
R7	Female			
R8	Female	35–40	2	Large number of COVID-19 patients
R9	Male	55–60	28	
R10	Female	45–50	12,5	
R11	Male	55–60	26	
R12	Male	35–40	8	Large number of COVID-19 patients
R13	Male	55–60	25	Large number of COVID-19 patients
R14	Female	50–55		
R15	Female	60–65	34	Risk group

### Theme i: uncertainties GPs faced during COVID-19

Interviewees mentioned they had to deal with general uncertainties regarding the new COVID-19 virus as little was known about the virus, its treatment and consequences. Moreover, GPs had to care for patients they did not know, and about whom they were not familiar with the medical and social background.

The uncertainties due to COVID-19 came across in all interviews. For example: what do symptoms of COVID-19 look like? When is someone in need of hospital referral? And, what are the benefits of hospital admission? Interviewees did not know what they were dealing with exactly, and what best care was. Furthermore, some of the interviewed GPs experienced difficulties in communication with their patients. They felt impeded to give clear information, as they themselves lacked knowledge due to the uncertainties regarding COVID-19. “What I know today, can be different in two days.” (R10).

Moreover, as patients in Dutch GP practices were divided between ‘clean’ COVID-19 free and contaminated practices, GPs had to care for patients they did not know. This introduced extra uncertainties, as they often were unfamiliar with patient’s background. Questions like ‘will he benefit from hospital referral’ or ‘will it harm him? What are his wishes and expectations of life?’ popped up. “Yes, [it made a difference, ed.] a lot. You can rely on the past. The relationship is better, people know who you are as a doctor. Perhaps because of that, there is also more trust. (…) So it does make things much easier. You know people better, what they are capable of. And you know a bit about how they live their life.” (R1) Some interviewees mentioned that not having a care history with patients caused complications in decision-making. One interviewee voiced this as follows: “That makes it way more complicated, and then you can get more knotted up with yourself. What does she actually want? Yes or no? And you also have to deal with the medical treatment. Maybe she will be fine, maybe she won’t.. But what does or doesn’t she want? (…) So you are aware of the fact that the chance to make a wrong decision is bigger.” (R4).

### Theme ii: the patient’s self-determination versus paternalism (ethical dilemma i)

This ethical dilemma consists of two conflicting moral values: the patients’ self-determination versus GP paternalism. The principle of respect for the patient’s autonomy is associated with enabling her or him to make her own decisions about receiving certain medical care. [32] Paternalism is ‘the intentional overriding of one person’s known preferences or actions by another, where the person who overrides justifies the action by the goal of benefiting or avoiding harm to the person whose preferences or actions are overridden.’ [33]

Some interviewed GPs claimed that the principle of self-determination guided decisions regarding hospital referral during COVID-19, regardless of the opinion of the GP. “I have to be absolutely certain before telling someone, you know. ‘it doesn’t make sense to go to the hospital, you simply should not go. And even then: if someone really wants to go, I am willing to support her or him.” (R2) However, because of the uncertainties due to COVID-19 and resulting lack of information on the disease, interviewees found it particularly complicated to interfere with the patients’ choices. “If I am not absolutely certain about whether someone will benefit from it, but I do take away her of his self-determination, I think that is absolutely.. no I think that is a no go. (…) Taking away someone’s self-determination in case of uncertainty? No.” (R2) Apparently, according to some, respecting the patient’s autonomy is increasingly important in times of uncertainties. When interviewees were not absolutely certain about the pros and cons of hospital referral, they felt it was not right to decide for the patient. They felt they had to act with certainty although the situation was uncertain.

However, this also depended on the situation. One interviewee explained it as follows: “Then (if a patient would like treatment, ed.) the patient is the asking party. When I suggest treatment and the patient does not want it, the patient is the refusing party. That is the difference.” (R13) The GPs’ gatekeeping role became critically important with regard to the ‘asking’ side, as the pressure on our hospitals increased. One of the interviewees stated that “I think a part of the fact the amount of IC-beds remained feasible, and the subsequent fact that hospitals were able to manage by pulling out all the stops, is because of the GP. That the GP did not refer patients who did not need to be referred to the hospital.” (R14) In other words: the gatekeeping function was deployed in order to balance against societal pressures.

According to the majority of interviewees, the ‘refusing party’ of patients not in favor of hospital referral existed due to fear. They were afraid they would be contaminated with COVID-19 in the hospital. Many interviewees stated they had to convince these patients about the fact they would benefit from hospital care. In certain cases however, interviewees felt they had to override patients refusing this, by acting more paternalistic. “Sometimes you even had to convince people: ‘You really need to go to the hospital!’. Because, people did not want to as they were afraid to pick up COVID in the hospital.” (R6).

In both cases, when the patient wants to get a hospital referral when it is not an adequate option and in the case the patient is too afraid to visit the hospital, some GPs felt they had to decide themselves instead of their patient. Moreover, acting more paternalistic was sometimes even seen as part of their job. For example, one GP stated that: “At some point, you just have to say ‘Your child is not that ill, we are not going to do this.’ And yes.. We are also educated to, in such cases, nót do it. Then you explain that a few times, and at a certain moment it suffices.”

### Theme iii: GP’s duty of care versus the autonomy rights of the GP (ethical dilemma ii)

This ethical dilemma derived from the high contagiousness of COVID-19, and the risk of being infected. This created a conflict between the GPs’ duty to treat and his or her own right to autonomy. The former is the principle that ‘a physician shall be dedicated to provide competent medical care, with compassion and respect for human dignity and rights.’ [34] The physician’s right to autonomy entails the fact the human behind the medical professional has moral rights and inherent dignity. [35] The tension between duty of care and autonomy can become an ethical dilemma when care provision is hazardous.

The feeling of owing a duty came across in the majority of the interviews. “That might sound a bit melodramatic, but I did experience it as a duty. It wasn’t a war, but you are the one who, based on your profession, obliged to be there and to do what you have to do. Because if you all refuse to do so, nothing will happen.” (R13) Some of the GPs even described this duty as a mission: “You also feel it is sort of a ‘vocation’ to be part of it.” (R8) Many GPs stated that this feeling increased during the crisis. However, because of the high contamination hazard of COVID-19, this duty was experienced differently among interviewees. Some argued that “And I think when a GP, in the midst of a crisis, is afraid of COVID her- or himself and because of that does things differently, that’s should simply be called unprofessional.”(R7) Others, especially during the beginning of COVID-19, experienced “I do have my own boundaries, but no-one asked about them.” (R15).

This conflict was mostly present when it concerned GPs’ loved ones. One of the interviewees was pregnant and did not know the risks for her baby when she would work with COVID-19 patients. “Yes, absolutely, I thought that [being at risk, red.] was difficult indeed. But also protecting yourself, that I thought, you know.. I don’t feel like actively pursuing this. (…) Because, I am pregnant at the moment.” (R3) GPs who were part of the group at risk, avoided contact with patients with COVID-19 symptoms. Instead, their colleagues were seeing these patients. On the other hand, some GPs who were at risk decided that their duty to care weighed more heavily than preventing their own risk. “For me personally, yes. I know I am in the risk zone. I am not naïve about the fact I could get seriously ill from it. But I will just accept that and just do whatever I can, and that’s it. And if it goes wrong, despite the fact I am doing what I can, than that’s that.” (R7) And “Look, if you have an outbreak of a contagious disease, if you are a doctor, yes: you still have to. You have to treat. I think every doctor would. Even if it is dangerous sometimes.” (R4) Most of the GPs in this study felt the duty to help as stated by one of them as follows: “I never considered to say: I report sick for the next month, they have to do it without me. It felt too important to be there, because they really need you.” (R8) The duty of care principle thus seemed to have a considerable weight.

### Theme iv: duty of care versus adequate care provision (ethical dilemma iii)

During the COVID-19 peak, patients were divided over newly composed ‘clean’ and ‘contaminated’ general practices. As a consequence, many interviewees had to care and make decisions for patients they did not know. This created a conflict between their duty of care and adequate care provision. The duty of care is described as: ‘a physician shall, in the provision of appropriate patient care, except in emergencies, be free to choose whom to serve, with whom to associate, and the environment in which to provide medical care’. [34] On the other hand, the physician has to provide adequate medical care, which entails the obligation to make access to an adequate level of care available to all, and for which physicians should advocate. [34].

The majority of interviewees claimed it was difficult to make decisions for unknown patients, indicating the importance of a trust relationship, but felt the duty to provide medical care. “It of course remains difficult to make decisions about people you don’t know, but refusing it never crossed my mind.” (R13) And “But I would do it [making decisions about patients she does not know, ed.], you know. Because, eventually you have to do something. But I am aware of the fact you can more easily make mistakes.” (R4) Most GPs in this study preferred their decisions to be based on the wishes and expectations of their patients. Many interviews felt uncomfortable making decisions regarding patients they did not had an established relationship with, and some of them even refused to decide about hospital referral.

## Discussion

In this study, it was explored how Dutch GPs experienced their role as a gatekeeper and which ethical dilemmas they faced when making decisions about hospital referrals in times of the first wave of the COVID-19 pandemic, both for COVID and non-COVID suspected patients. One overarching core theme and three ethical dilemmas were identified. The core theme concerned (1) uncertainties caused by COVID-19. It overarched three themes of ethical dilemmas, which became (more) prominent during the COVID-19 crisis: (2) the patients’ self-determination vs. the GP’s paternalism, (3) the GP’s duty of care vs. the GP’s autonomy rights, and (4) the GP’s duty of care vs. adequate care provision.

### Ethical dilemma i: self-determination versus paternalism

During the COVID-19 crisis, many GPs experienced decision making as problematic. Instead of SDM, they more often had to choose between conflicting values: self-determination of the patient and their own paternalism, which was experienced as an ethical dilemma. GPs in this study considered paternalism not justified, because of the lack of knowledge about the pros and cons of hospital referral. Many GPs stated that despite these uncertainties, it was expected of them to make clear decisions, to paternalize. Numerous GPs found this difficult: to be obliged to act as if being certain in an uncertain situation. This lack of knowledge also existed when the GP would let the patient decide. Due to these uncertainties, SDM was challenging during the COVID-19 crisis.

Many GPs made a differentiation between the patient being the asking party and the patient as refusing party. If the patient *asked* for hospital referral, the GP’s gatekeeper role became enormously important due to the fact the pressure on our healthcare system and hospitals increased. Fair allocation is hugely relevant in the midst of scarcity [36], and health care professionals experience an important role in controlling COVID-19 and therefore sometimes refuse treatment [7]. On the other hand, interviewees in our study had to override patients’ *refusal* of a hospital admission (outpatient or inpatient) in certain cases. This finding is in line with previous research indicating that overriding patients’ wishes might benefit the patient, especially when the patient is the refusing party [37, 38].

### Ethical dilemma ii: duty of care versus GPs’ autonomy rights

Most GPs interviewed in this study stated they felt a tension between their duty of care as a professional versus their own risk of getting infected with COVID-19. However, their duty of care became more explicit, like a mission, during the pandemic. This value overshadowed the fear for their own health, even when belonging to a risk group themselves. This confirms recent COVID-19 studies [35, 39–41]. But are there limits to the duty of care? And what do they look like? In a study regarding heroic language used for healthcare workers and their work, it is stated that the duty of care is limited, even during crises like the COVID-19 pandemic [42]. This is being illustrated by the fact it was advised to avoid contact with patients who were having health problems that made them as professional vulnerable for complications of COVID-19. Numerous interviewees in our study also stated they covered for colleagues at risk, showing the limits to their duty of care. Others however were at risk themselves but still decided to work as a GP during the COVID-19 pandemic.

The difficulty of grounding the duty of care is consistent with former research, showing it is a social contract with society [43]. However, it is important to define the limits of the duty of care [42]. Many participating GPs experienced dilemmas regarding the duty of care towards their patients and their own responsibility for not contaminating loved ones. This conflict confirms an opinion paper on exactly this topic [44]. Because of the many uncertainties during COVID-19, it is important to have more clarity about limits to the duty of care. We recommend further research on this dilemma.

### Ethical dilemma iii: duty of care versus adequate care provision

Although adequacy of care is difficult to define, i.e. is not a ‘black and white’ issue, interviewees in this study experienced difficulties in making decisions regarding referring patients when it concerned patients they did not yet know. They stated that, because of their duty of care, they would make such decisions, but felt impeded to provide the care they wished to provide. Although GPs are accustomed to make decisions for patients they do not have a care history with when on duty at the out-of-hours service, it was during the COVID-19 period that this became a dilemma. Clearly, a trustful relationship between patient and physician is important when it concerns serious problems and decision making [45], like during this pandemic. This ethical dilemma also needs to be taken into consideration for future pandemics.

### Strengths and weaknesses

This is one of the first studies about the COVID-19 crisis that concerned professionals in general practice; most literature focused on hospitals and nursing homes. Despite the lock-down, we managed to interview sufficient GPs in a short period of time. However, GPs who worked in regions with less victims had not always experienced dilemmas in their work; their input was partly based on anticipating on the realistic option of having to deal with such dilemmas in the near future. Moreover, although this would have been valuable information, a comprehensive overview of reasons for non-participation was not systematically collected. Next, the female interviewees were younger and had less working experience than the male ones, which might have influenced our study’s level of generalizability.

## Conclusion

In conclusion, this study identified three ethical dilemmas experienced by Dutch GPs when making decisions about referring patients to the hospital, in times of the first wave of the COVID-19 pandemic, both for COVID and non-COVID suspected patients. These ethical dilemmas are well-known dilemmas in general practice but became more prominent during the COVID-19 crisis, because of lack of knowledge about the virus, risk for the GP to infect beloved ones, scarcity of hospital beds, and loneliness of the patient during hospital admission. When developing guidelines and protocols for crisis management like this pandemic, these ethical dilemmas should be taken into account.

## Data Availability

The anonymized dataset used during the current study is available from the corresponding author on reasonable request.

## Appendix 1: Topic guide

- Question 1: Which challenges did you experience during the COVID-19 pandemic? (concerning hospital referrals).
- Prompts: How do you see your own gatekeeper role? How do challenges experienced relate to pre-COVID times?

- Question 2: When were the decisions concerning hospital referral during the COVID-19 pandemic really simplified?
- Prompts: Did uncertainty during the pandemic influence this? If so: how?

- Question 3: In what way were your own moral considerations important in deciding about hospitalization during the COVID-19 pandemic?
- Prompts: Could you elaborate on them? (How) do you think they can or should play a role in your decision-making as a GP?

## Abbreviations

COVID-19: Coronavirus disease 2019
ACP: Advance care planning
GP: General practitioner
COPD: Chronic obstructive pulmonary disease
EHR: Electronic health record
ICU: Intensive care unit
